# High-density lipoprotein cholesterol level as an independent protective factor against aggravation of acute pancreatitis: a case–control study

**DOI:** 10.3389/fendo.2023.1077267

**Published:** 2023-12-06

**Authors:** Qingqiang Ni, Zetao Yu, Peng Zhang, Hongtao Jia, Fangfeng Liu, Hong Chang

**Affiliations:** ^1^ Department of Hepatobiliary Surgery, Shandong Provincial Hospital Affiliated to Shandong First Medical University, Jinan, Shandong, China; ^2^ Department of Hepatobiliary Surgery, Shandong Provincial Hospital, Shandong University, Jinan, Shandong, China; ^3^ Intensive Care Unit (ICU), Shandong Provincial Hospital Affiliated to Shandong First Medical University, Jinan, Shandong, China

**Keywords:** acute pancreatitis, high-density lipoprotein cholesterol, intensive care unit, case-control studies, protective factor

## Abstract

**Background and aims:**

At present, evidence on the association between high-density lipoprotein cholesterol (HDL-C) levels and aggravation of acute pancreatitis (AP) is limited. This study aimed to investigate the relationship between the lowest HDL-C level during intensive care units (ICU) stay and AP aggravation and to determine the optimum cutoff lowest HDL-C level.

**Methods:**

Patients admitted to the ICU of the Shandong Provincial Hospital for AP from 2015 to 2021 were included. The lowest HDL-C level during ICU stay was set as the independent variable, and the progression or non-progression to severe AP (SAP) was set as the dependent variable. Univariate and multivariate analyses were performed to determine the relationship between the two variables, and receiver operating characteristic (ROC) curves were plotted to analyze the predictive ability of the lowest HDL-C level for progression to SAP.

**Results:**

This study included 115 patients. The difference in the lowest HDL-C level between the SAP and moderately SAP groups was significant (*P* < 0.05). After adjusting for covariates, the lowest HDL-C level showed a negative correlation with the occurrence of SAP, with a relative risk of 0.897 (95% confidence interval: 0.827–0.973). The area under the ROC curve for prediction of AP aggravation by the lowest HDL-C level was 0.707, and the optimum cutoff lowest HDL-C level was 0.545 mmol/L.

**Conclusion:**

No less than 0.545 mmol/L of the HDL-C level during ICU stay may be an independent protective factor for the aggravation of AP.

## Introduction

Acute pancreatitis (AP) is an inflammatory disease characterized by abnormal activation of pancreatic digestive enzymes, with varying etiologies, that exert digestive effects on the pancreas and peripheral organs. AP mainly manifests as local or systemic inflammation, and its incidence has been steadily increasing worldwide (1). According to the Atlanta classification system, AP can be classified as mild AP (MAP), moderately severe AP (MSAP), or severe AP (SAP) (2, 3). Patients with MAP present with mild symptoms, a short disease course of 1 week, and self-limiting attacks. Approximately 20% of cases progress to SAP, with persistent organ failure (POF), which is the main cause of death in patients with SAP. Considering that the mortality rate of SAP is approximately 20%–40%, early intervention and prediction of AP aggravation are of utmost importance (4–6).

To assess the severity of AP and for early prediction of AP, the Ranson criteria, Acute Physiology and Chronic Health Evaluation II score, bedside index for severity in acute pancreatitis, and Balthazar grades have been reported (7–10). However, scoring is relatively complex and of lower practicality in clinical practice, thereby necessitating simple hematological indicators for predicting AP progression. Procalcitonin (PCT) level, interleukin-6 (IL-6) level, white blood cell (WBC) count, and macrophage migration inhibitory factor level have been successively identified as indicators of AP progression and have shown promising results in small sample size studies, but the effectiveness and practicality of these indicators remain to be explored (11–13). High-density lipoprotein cholesterol (HDL-C), mainly secreted by the liver and small intestine, plays an important role in maintaining cholesterol homeostasis. The relationship between HDL-C level and the occurrence of cardiovascular disease has been widely demonstrated (14, 15). HDLs participate in the immune response of the body via their ability to modulate cholesterol bioavailability in immune cells (16). Therefore, they serve an indispensable role in inhibiting the inflammatory response (17). HDL-C also inhibits the expression of adhesion molecules by transferring miR223 to receptor endothelial cells, thereby achieving anti-infection effects. miR223 is the most abundant miRNA in monocytes and macrophages and plays a key role in the regulation of the inflammatory response (18, 19).

Many recent studies have reported the relationships of HDL-C level with POF and the severity of AP (20–26). However, most of them have focused on HDL-C level within 24 h of admission for the prediction of AP-related patient condition rather than exploring the relationship between subsequent changes in HDL-C level and the occurrence of SAP. Therefore, the present study aimed to investigate the relationship between the lowest HDL-C level of patients with AP during intensive care units (ICU) stay and the progression to SAP and to determine the predictive value of the lowest HDL-C level for AP aggravation.

## Methods

### Study design

This case–control study was performed to explore the relationship of the lowest HDL-C level during hospital stay with the onset and development of AP in patients with MSAP or SAP and to determine the optimum cutoff lowest HDL-C level for the prediction of SAP. The lowest HDL-C level measured during ICU stay was set as the independent variable, and the occurrence or non-occurrence of SAP was set as the dependent variable. Our study was conducted in accordance with the Strengthening the Reporting of Observational Studies in Epidemiology guidelines.

### Study population

**Figure 1:**
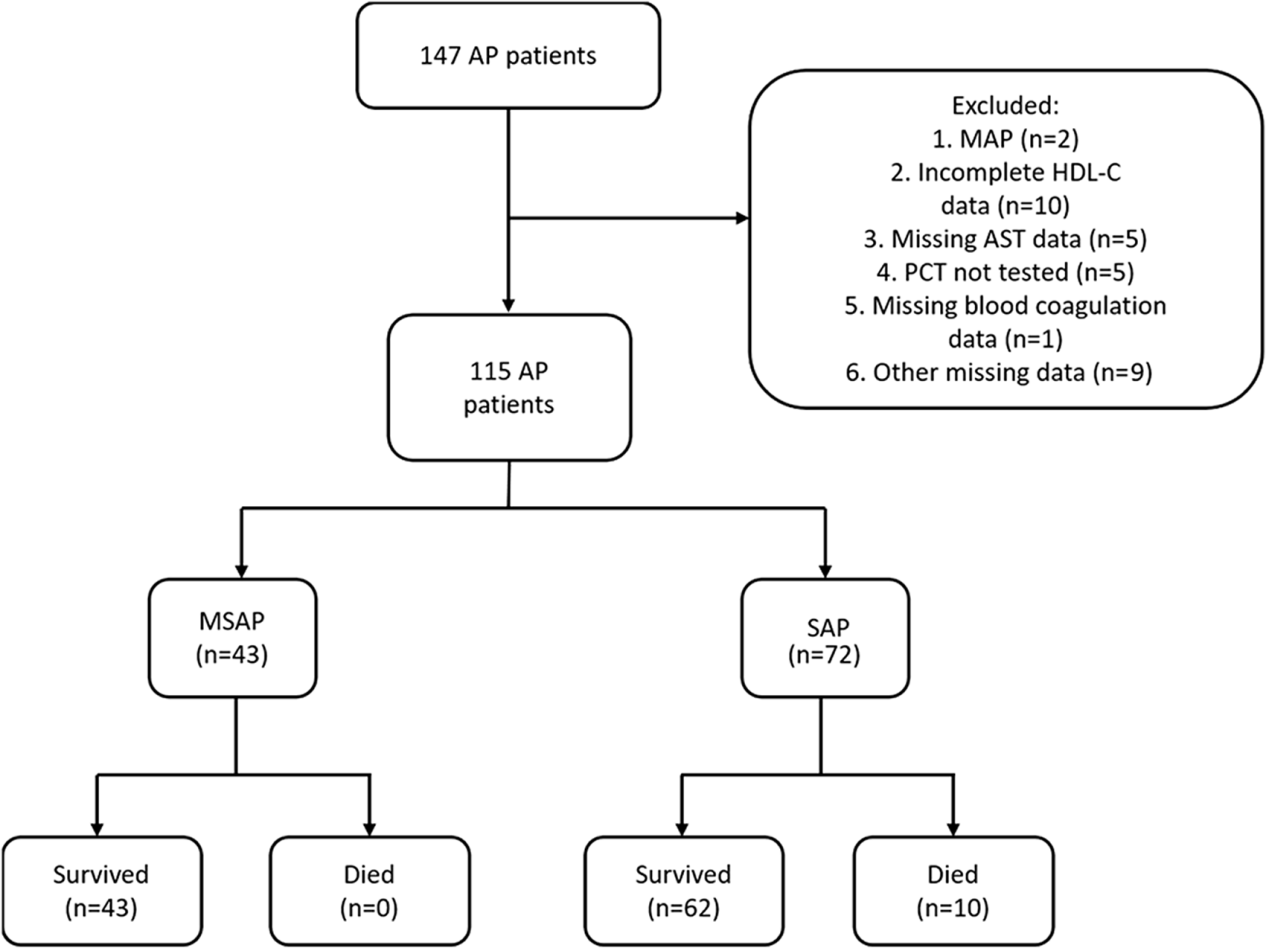
Flowchart of the inclusion process of study patients. A total of 115 patients with AP were included.

We obtained the data of 147 patients with AP admitted to the ICU at the Shandong Provincial Hospital from 2015 to 2021. AP was diagnosed when any two of the following criteria were fulfilled: (1) abdominal pain suggestive of AP; (2) the serum lipase or amylase level exceeding thrice the upper limit of the normal range; and (3) computed tomography findings suggestive of AP (27). The severity of AP was classified using the Atlanta classification system. Considering the small number of patients with MAP during the study period, only those with MSAP or SAP were included. We excluded 10 patients due to incomplete HDL-C data, five patients with missing aspartate transaminase (AST) data, five patients who did not undergo PCT measurement, two patients with MAP, one patient with missing blood coagulation data, and nine patients with other missing data (**Figure 1**).

Data were collected using the electronic health record search system at the Shandong Provincial Hospital. The Ethics Committee waived the requirement of obtaining informed consent because of the retrospective study design. To protect patient privacy, personal information of the patients was not included as part of the study data. This retrospective study was approved by the Ethics Committee of Shandong Provincial Hospital before commencement and was conducted in accordance with the principles of the Declaration of Helsinki.

### Variables

The following data were obtained from the health records of the patients during their ICU stay: age; sex; etiology of AP; interval between symptom onset and hospital admission; presence/absence of diabetes mellitus; vital signs (body temperature, pulse rate, and systolic blood pressure); results of hematological testing, including WBC count, the highest WBC count, red blood cell [RBC] count, hemoglobin level, hematocrit level, platelet count, lymphocyte count, monocyte count, neutrophil count, neutrophil/lymphocyte ratio, monocyte/lymphocyte ratio, platelet/lymphocyte ratio, red cell distribution width [RDW-CV], mean platelet volume [MPV], AST level, alanine transaminase [ALT] level, gamma-glutamyltransferase [GGT] level, alkaline phosphatase [ALP] level, albumin level, globulin level, albumin/globulin ratio, total bilirubin [TBIL] level, direct bilirubin [DBIL] level, indirect bilirubin [IBIL] level, glucose level, PCT level at hospital admission, highest PCT level, amylase level, blood urea nitrogen [BUN] level, creatinine level, BUN/creatinine ratio, calcium level, phosphorus level, potassium level, sodium level, chloride level, prothrombin time (PT), fibrinogen (FIB) level, activated partial thromboplastin time (APTT), D-dimer level, highest triglyceride (TG) level, highest total cholesterol (TC) level, highest HDL-C level, and highest low-density lipoprotein cholesterol (LDL-C) level; history of smoking and drinking; and presence/absence of abdominal pain during onset. Except the highest values specified for certain parameters, all data were recorded at hospital admission.

### Definitions

Respiratory, cardiovascular, and renal organ failures were assessed using the Marshall scoring system. Organ failure was diagnosed when more than two organs in any system were affected, and POF was defined as organ failure exceeding 48 h. Two experienced doctors classified the patients based on the severity of organ failure using the Atlanta classification system.

### Data analysis

Categorical variables are expressed as frequency (percentage). Normally distributed continuous variables are expressed as mean ± standard deviation, and non-normally distributed continuous variables are expressed as median (interquartile range). Univariate analyses were performed using the Fisher exact test, Student’s *t*-test, and Mann-Whitney U test for the above-mentioned three types of variables, and the multivariate analysis was performed using Poisson regression. To investigate the relationship between the lowest HDL-C level during ICU stay and the progression of MSAP to SAP in patients with MSAP or SAP and to determine the optimum cutoff lowest HDL-C level, the following steps were adopted: (1) patients were categorized into the MSAP or SAP group, and univariate analysis was performed for all the variables, with *P* < 0.05 set as statistical significance in the two-tailed test; (2) significant variables in the univariate analysis were included in the multivariate analysis using Poisson regression to determine the independent risk factors for AP aggravation, with *P* < 0.05 set as statistical significance, and the relative risk (RR) and 95% confidence interval (CI) were calculated; and (3) receiver operating characteristic (ROC) curves were plotted and analyzed to obtain the optimum cutoff, sensitivity, specificity, and Youden index values to evaluate the predictive ability of the lowest HDL-C level for SAP and determine the optimum cutoff lowest HDL-C level for the prediction of SAP. Statistical analyses were performed using SPSS 26.0 (IBM Corp, Armonk, New York).

## Results

### Baseline characteristics of the study population

A total of 115 patients with AP were included. The MSAP group comprised 43 patients, seven (16.3%) of whom were diabetic. The most common etiologies of AP were hypertriglyceridemia and idiopathic (37.2% each), followed by alcoholism (20.9%) and biliary (4.7%). The SAP group comprised 72 patients; among these, nine (12.5%) patients were diabetic, and the etiologies were alcoholism (23.6%), biliary (18.1%), hypertriglyceridemia (13.9%), and idiopathic (44.4%). **Tables 1**–**3** present the baseline characteristics of the 115 patients. The two groups showed no significant differences in the serum amylase level, heart rate, respiratory rate, pyrexia, diabetes, WBC count, RBC count, hemoglobin level, hematocrit level, lymphocyte count, monocyte count, neutrophil count, neutrophil/lymphocyte ratio, RDW-CV, ALP level, albumin level, globulin level, albumin/globulin ratio, glucose level, BUN/creatinine ratio, calcium level, phosphorus level, potassium level, chloride level, FIB level, and APTT. As shown in **Table 4**, the lowest HDL-C value was most distributed in the range of 0.26-0.50 mmol/L, with a total of 41 patients, accounting for 35.7% of the total number. The incidence of SAP progression from high to low was 0-0.25 mmol/L, 0.26-0.50 mmol/L, 0.51-0.75 mmol/L, >1.00 mmol/L and 0.76-1.00 mmol/L, respectively.

**Table 1:** Baseline patient characteristics (categorical data based on differences analyzed using the chi-square test).

Characteristic	MSAP	SAP	*P*-value
Men	20(46.5%)	50(69.4%)	0.018*
Pyrexia at intensive care unit admission	14(32.6%)	33(45.8%)	0.176
Diabetes	7(16.3%)	9(12.5%)	0.588
Smoking	16(37.2%)	32(44.4%)	0.558
Drinking	16(37.2%)	35(48.6%)	0.251
Abdominal pain during onset	42(97.7%)	72(100%)	0.374

*significant difference (P < 0.05).

All characteristics are categorical variables expressed as n (%) and statistically analyzed using the Fisher exact test.

**Table 2:** Baseline patient characteristics (normally distributed continuous variables based on differences analyzed using the Student’s *t*-test).

Characteristics	MSAP	SAP	*P*-value
RBC (10^12^/L)	4.28±0.80	3.99±0.97	0.083
HGB (g/L)	128.56±26.53	121.92±30.01	0.233
MPV (fl)	10.52±1.11	11.20±1.44	0.009*
TP (g/L)	55.32±5.09	55.30±8.98	0.989
ALB (g/L)	31.02±6.29	31.97±5.89	0.417
A/G	1.31±0.30	1.43±0.38	0.064
Ca (mmol/L)	1.90±0.30	1.88±0.34	0.705
FIB (g/L)	6.42±2.03	6.03±2.36	0.361

*significant difference (P < 0.05).

All characteristics are normally distributed continuous variables expressed as mean ± standard deviation and statistically analyzed using the Student’s t-test.

RBC, red blood cell; HGB, hemoglobin; MPV, mean platelet volume; TP, total protein; ALB, albumin; A/G, albumin/globulin ratio; Ca, calcium; FIB, fibrinogen.

**Table 3:** Baseline patient characteristics (non-normally distributed continuous variables; differences analyzed using the rank sum test).

Characteristic	MSAP	SAP	*P*-value
Age (years)	35.00 (29.00,41.00)	44.50 (35.50,66.50)	<0.001*
WBC (10^9^/L)	12.81 (9.02,17.06)	13.25 (10.02,18.37)	0.686
Highest WBC count (10^9^/L)	16.17 (12.27,22.92)	20.02 (15.71,26.40)	0.008*
HCT (%)	38.70 (33.30,43.10)	36.85 (29.68,42.88)	0.290
PLT (10^9^/L)	235.00 (185.00,296.00)	171.50 (99.50,233.50)	<0.001*
LYMPH (10^9^/L)	1.06 (0.69,1.52)	0.93 (0.65,1.48)	0.384
MONO (10^9^/L)	0.56 (0.41,0.98)	0.71 (0.39,0.94)	0.395
NEUT (10^9^/L)	11.09 (7.50,14.03)	10.84 (8.38,15.38)	0.597
NLR	10.22 (7.23,17.32)	11.17 (6.35,20.98)	0.631
MLR	0.48 (0.37,0.92)	0.65 (0.47,1.05)	0.025*
PLR	225.26 (151.31,292.39)	147.58 (110.59,222.02)	0.005*
RDW-CV (%)	13.90 (13.40,14.60)	13.85 (13.43,14.58)	0.742
AST (U/L)	23.00 (16.00,31.00)	54.00 (28.00,104.50)	<0.001*
ALT (U/L)	13.00 (8.00,34.00)	37.00 (17.25,97.75)	<0.001*
GGT (U/L)	39.00 (21.00,109.00)	52.00 (31.50,157.25)	0.035*
ALP (U/L)	80.00 (62.00,123.00)	72.50 (53.25,100.00)	0.164
PA (mg/L)	110.00 (71.00,148.30)	105.50 (78.30,146.78)	0.835
GLO (g/L)	24.30 (20.10,27.30)	23.00 (19.70,26.08)	0.128
TBIL (μmol/L)	18.30 (11.60,25.50)	25.55 (15.91,52.63)	0.002*
DBIL (μmol/L)	3.80 (2.60,6.00)	7.51 (4.72,22.28)	<0.001*
IBIL (μmol/L)	13.10 (8.70,19.70)	16.28 (10.91,24.93)	0.045*
GLU (μmol/L)	9.03 (6.94,13.76)	9.61 (6.97,13.14)	0.688
BUN (mmol/L)	4.90 (3.70,6.80)	10.30 (5.80,15.35)	<0.001*
CREA (μmol/L)	58.90 (43.27,78.00)	98.65 (53.10,217.73)	<0.001*
BUN/CREA	82.60 (60.00,101.9)	90.85 (60.50,110.95)	0.495
P (mmol/L)	0.73 (0.49,0.97)	0.76 (0.43,1.04)	0.817
K (mmol/L)	3.99 (3.60,4.50)	4.03 (3.60,4.49)	0.817
Na (mmol/L)	136.00 (132.20,138.00)	139.25 (134.00,143.83)	0.001*
CL (mmol/L)	104.40 (99.90,107.00)	104.75 (100.00,109.98)	0.440
APTT (s)	38.20 (32.50,42.20)	39.15 (35.48,47.30)	0.129
PT (s)	14.5 (13.5,15.7)	15.55 (14.60,17.38)	0.003*
D-dimer (mg/L)	4.86 (2.26,6.96)	6.62 (3.70,10.82)	0.004*
PCT level at admission (ng/mL)	0.88 (0.33,1.87)	2.75 (1.08,14.31)	<0.001*
Highest PCT level (ng/mL)	1.19 (0.48,2.21)	6.51 (1.52,36.24)	<0.001*
Highest TG level (mmol/L)	5.37 (2.42,11.89)	3.53 (1.84,6.35)	0.015*
Highest TC level (mmol/L)	6.75 (4.31,9.78)	1.84 (3.53,6.35)	0.001*
Lowest HDL-C level (mmol/L)	0.63 (0.50,0.81)	0.46 (0.30,0.60)	<0.001*
Highest LDL-C level (mmol/L)	3.84 (2.72,5.24)	2.78 (2.10,3.79)	<0.001*
AMS (U/L)	162.00 (70.00,519.00)	163.00 (74.25,487.50)	0.809
Heart rate	107.00 (90.00,123.00)	111.50 (99.25,128.75)	0.194
Respiratory rate	21.00 (19.00,27.00)	21.00 (18.00,30.00)	0.945

*significant difference (P < 0.05).

All characteristics are non-normally distributed continuous variables expressed as median (interquartile range) and statistically analyzed using the Mann-Whitney U test.

NLR, neutrophil/lymphocyte ratio; MLR, monocyte/lymphocyte ratio; PLR, platelet/lymphoc yte ratio; AST, aspartate transaminase; ALT, alanine transaminase; GGT, gamma-glutamyltransferase; ALP, alkaline phosphatase; BUN, blood urea nitrogen; CREA, creatinine; PCT, procalcitonin; TG, triglyceride; TC, total cholesterol; LDL-C, low-density lipoprotein cholesterol; HDL-C, high-density lipoprotein cholesterol; WBC, white blood cell; HCT, hematocrit; PLT, platelet; LYMPH, lymphocyte; MONO, monocyte; NEUT, neutrophil; RDW-CV, red cell distribution width; PA, prealbumin; GLO; globulin; TBIL, total bilirubin; DBIL, direct bilirubin; IBIL, indirect bilirubin; GLU, glucose; BUN/CREA, BUN/creatinine ratio; P, phosphorus; K, potassium; Na, sodium; APTT, activated partial thromboplastin time; PT, prothrombin time; PCT, procalcitonin; AMS, serum amylase.

**Table 4:** Distribution of HDL-C levels and incidence of SAP.

HDL-C range (mmol/L)	MSAP+SAP (%)	SAP (%)
0—0.25	12 (10.4%)	11 (91.7%)
0.26—0.50	41 (35.7%)	31 (75.6%)
0.51—0.75	38 (33.0%)	20 (52.6%)
0.76—1.00	13 (11.3%)	5 (38.5%)
>1.00	11 (9.6%)	5 (45.5%)

### Univariate analysis

The MSAP group had 20 (46.5%) men and 23 (53.5%) women, with a median age of 35.00 (range: 29.00–41.00) years. The SAP group had 50 (69.4%) men and 22 (30.6%) women, with a median age of 44.50 (33.50–66.50) years (**Tables 1** **–** **3**). The serum amylase level, heart rate, respiratory rate, pyrexia, diabetes, WBC count, RBC count, hemoglobin level, hematocrit level, lymphocyte count, monocyte count, neutrophil count, neutrophil/lymphocyte ratio, RDW-CV, ALP level, albumin level, globulin level, albumin/globulin ratio, glucose level, BUN/creatinine ratio, calcium level, phosphorus level, potassium level, chloride level, FIB level, or APTT was not significant (*P* > 0.05) in the univariate analysis. The highest WBC count, MPV, monocyte/lymphocyte ratio, AST, ALT, GGT, TBIL level, DBIL level, IBIL level, BUN level, creatinine level, sodium level, PT, D-dimer level, PCT level at hospital admission, and highest PCT level were positively correlated with SAP, whereas the platelet count, platelet/lymphocyte ratio, lowest HDL-C level, highest LDL-C level, and highest TG level were negatively correlated with SAP.

### Multivariate Poisson regression analysis

Age (RR: 1.002; 95% CI: 1.000–1.004) and the highest WBC count (RR: 1.004; 95% CI: 1.001–1.007) were independent risk factors for SAP, and HDL-C level exceeding the lowest HDL-C level during the ICU stay (RR: 0.897; 95% CI: 0.827–0.973) was an independent protective factor for SAP. Sex, MPV, monocyte/lymphocyte ratio, AST, ALT, GGT, TBIL level, DBIL level, IBIL level, BUN level, creatinine level, sodium level, PT, D-dimer level, PCT level at hospital admission, the highest PCT level, platelet count, platelet/lymphocyte ratio, the highest LDL-C level, and the highest TG level were not significant in the multivariate regression analysis (*P* > 0.05). **Table 5** presents the results of the multivariate Poisson regression analysis.

**Table 5:** Multivariate Poisson regression analysis.

Variable	RR (95% CI)	*P*-value
Age	1.002 (1.000-1.004)	0.020*
Sex	1.054 (0.981-1.131)	0.147
Highest WBC count (10^9^/L)	1.004 (1.001-1.007)	0.003*
PLT (10^9/L)	1.000 (0.999-1.000)	0.206
MLR	0.976 (0.919-1.036)	0.421
PLR	1.000 (1.000-1.000)	0.779
MPV (fl)	1.016 (0.995-1.038)	0.129
AST (U/L)	1.000 (1.000-1.000)	0.836
ALT (U/L)	1.000 (1.000-1.000)	0.660
GGT (U/L)	1.000 (1.000-1.000)	0.521
TBIL (μmol/L)	1.000 (0.997-1.004)	0.847
DBIL (μmol/L)	0.999 (0.994-1.004)	0.701
IBIL (μmol/L)	1.000 (1.000-1.000)	0.999
BUN (mmol/L)	0.999 (0.992-1.006)	0.781
CREA (μmol/L)	1.000 (1.000-1.000)	0.187
Na (mmol/L)	1.003 (0.997-1.008)	0.315
PT (s)	0.997 (0.987-1.007)	0.516
D-dimer (mg/L)	1.004 (0.999-1.009)	0.121
PCT level at admission (ng/mL)	1.000 (0.998-1.002)	0.882
Highest PCT level (ng/mL)	1.000 (0.999-1.001)	0.566
Highest TG level (mmol/L)	0.999 (0.994-1.004)	0.676
Highest TC level (mmol/L)	1.004 (0.976-1.032)	0.800
Lowest HDL-C level (mmol/L)	0.897 (0.827-0.973)	0.009*
Highest LDL-C level (mmol/L)	0.980 (0.941-1.021)	0.331

*statistically significant difference (P < 0.05).

MLR, monocyte/lymphocyte ratio; PLR, platelet/lymphocyte ratio; AST, aspartate transaminase; ALT, alanine transaminase; GGT, gamma-glutamyltransferase; BUN, blood urea nitrogen; CREA, creatinine; PCT, procalcitonin; TG, triglyceride; TC, total cholesterol; LDL-C, low-density lipoprotein cholesterol; HDL-C, high-density lipoprotein cholesterol; MPV, mean platelet volume; TBIL, total bilirubin; DBIL, direct bilirubin; IBIL, indirect bilirubin; PLT, platelet; PT, prothrombin time; PCT, procalcitonin.

### Predictive value of the lowest HDL-C level for SAP

**Figure 2:**
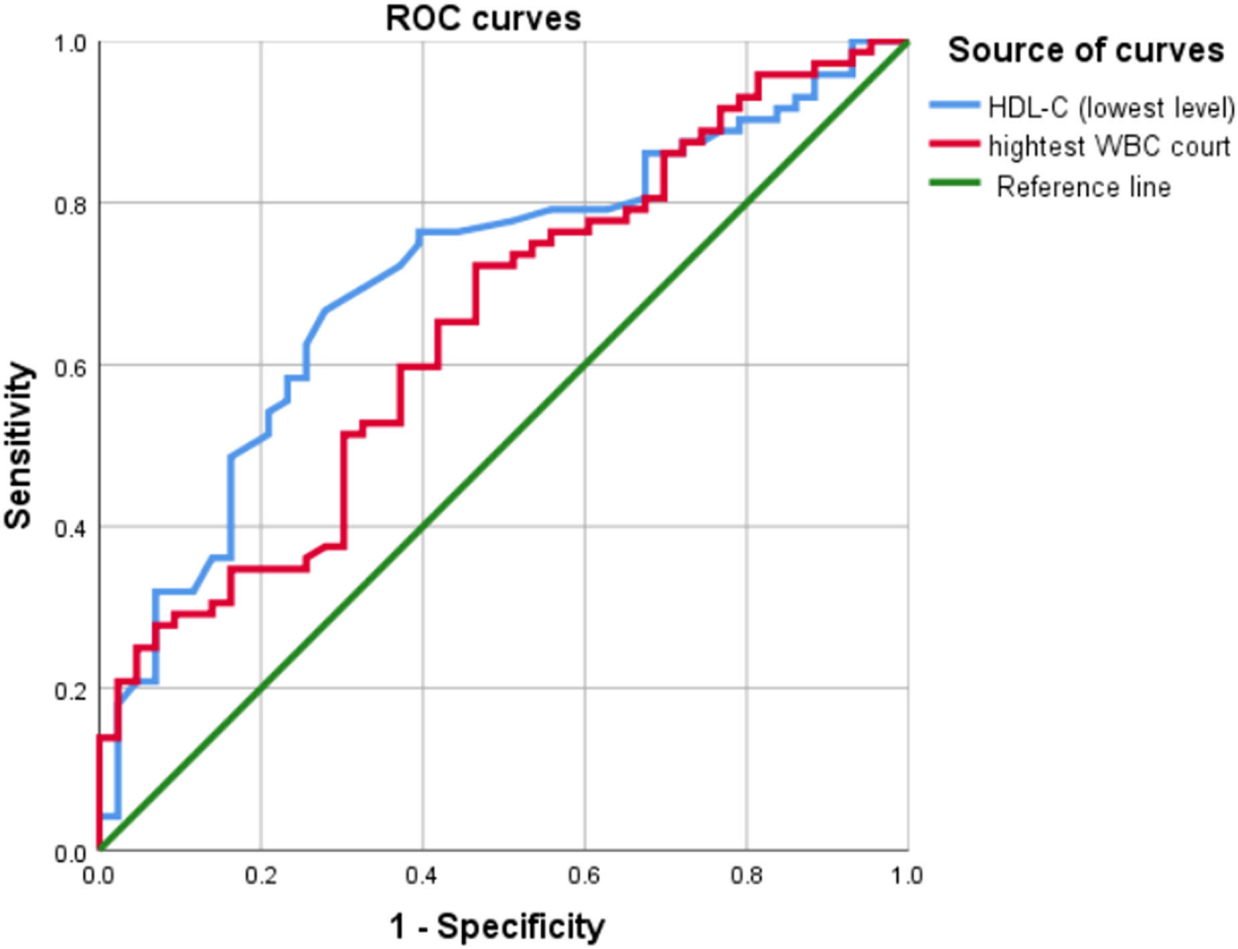
Results of the receiver operating characteristic curve analysis. ROC curves, receiver operating characteristic curves; WBC, white blood cell; HDL-C, High-density lipoprotein cholesterol.

The highest WBC count (95% CI: 0.545–0.750; area under the ROC curve [AUC] = 0.684) was a poor predictor of SAP, whereas the lowest HDL-C level during ICU stay (95% CI: 0.610–0.804; AUC = 0.707) was a better predictor of SAP, with Youden’s index of 0.388, optimum cutoff of 0.545 mmol/L, sensitivity of 0.667, and specificity of 0.721. **Table 6** shows the predictive value of the lowest HDL-C level and highest WBC count for SAP. **Figure 2** shows the ROC curves for progression to SAP.

**Table 6:** Results of severe acute pancreatitis prediction using receiver operating characteristic curves.

Variable	AUC	Threshold	Sensitivity	Specificity	Youden index	PLR	NLR
Lowest HDL-C level	0.707	0.545	0.667	0.721	0.388	2.390	0.462
Highest WBC count	0.648	16.735	0.722	0.535	0.257	1.553	0.520
PCT level at admission	0.737	2.025	0.611	0.791	0.402	2.923	0.491
Neutrophil granulocyte	0.530	8.12	0.778	0.349	0.127	1.195	0.636

PLR, positive likelihood ratio; NLR, negative likelihood ratio.

## Discussion

The study results indicate that HDL-C level higher than the lowest HDL-C level during ICU stay was an independent protective factor for the progression of MSAP to SAP in patients admitted to the ICU. The lowest HDL-C level was negatively correlated with progression to SAP, and an increase of 1 unit in the lowest HDL-C level caused a 0.897-fold change in the probability of progression to SAP. Among different lowest HDL-C levels, patients with lower HDL-C levels have a higher probability of developing SAP, and the incidence rate in the >1.00 mmol/L level is higher than that in the 0.76-1.00 mmol/L level. The reason for this deviation may be attributed to the limited number of patients included, especially in the >1.00 mmol/L range and the 0.76-1.00 mmol/L range. The optimum cutoff lowest HDL-C level for the prediction of progression to SAP was 0.545 mmol/L.

HDL-C is mainly synthesized in the liver and plays a key role in mediating the transport of cholesterol from surrounding tissues to the liver. It also exerts antioxidant, pro-fibrinolytic, and anti-thrombotic effects. A possible mechanism of the negative correlation between the lowest HDL-C level and the progression to SAP is as follows: progression of AP is closely associated with the systemic inflammatory response syndrome (28, 29); when inflammation occurs, the ability of HDL-C to neutralize toxic substances leads to the binding of massive amounts of endotoxins to HDL-C, thereby decreasing the HDL-C level (16, 30). In addition, the inflammatory cytokines IL-6 and tumor necrosis factor-α can inhibit HDL-C synthesis, whereas the acute phase protein secretory phospholipase A2 can accelerate HDL-C degradation (20). HDL-C also participates in innate immunity through innate host defense activity and regulates the participation of miR223 in the anti-infection process at the genetic level by transferring miR223 to receptor endothelial cells, which inhibits the expression of adhesive molecules (18, 19). Research has shown that inhibiting the NLRP3/Caspase-1 signaling pathway can alleviate pyroptosis and inflammatory response in hyperlipidemic pancreatitis models (31). HDL can decrease the expression of NLRP3 and its downstream inflammatory cytokines (32). Therefore, HDL may alleviate pyroptosis and inflammatory response in hyperlipidemic pancreatitis models by inhibiting the NLRP3/Caspase-1 signaling pathway. Studies have shown that activating the NRF2/HO-1-mediated ferroptosis pathway can improve acute pancreatitis, while oxidative stress damage and reduced expression of HDL can accompany the inhibition of the Nrf2/HO-1 pathway (33, 34). Therefore, HDL may alleviate acute pancreatitis by activating the NRF2/HO-1-mediated ferroptosis pathway. Studies have shown that the severity of acute pancreatitis can be improved by inhibiting the PI3K/Akt pathway (35). Inhibiting scavenger receptor class B type I (SR-BI) is sufficient to cause apoptosis, elevated intracellular reactive oxygen species levels, and decreased PI3K/AKT signaling (36). SR-BI is a versatile HDL receptor protein (37). Therefore, HDL may improve the severity of acute pancreatitis by inhibiting the PI3K/Akt pathway.

A study by Peng et al. involving 66 patients in the ICU revealed that the HDL-C level measured at ICU admission was predictive of the occurrence of POF and was negatively correlated with disease severity (24). However, the present study involved a larger number of patients and adopted a wider variety of hematological indicators as covariances, which would lead to greater accuracy in the multivariate analysis results. In another case–control study on the relationship between HDL-C level and severe pancreatitis by Li et al., the HDL-C level within 24 h of hospital admission was negatively correlated with the severity of pancreatitis (20). However, the aforementioned studies merely focused on the relationship between HDL-C level at hospital admission and SAP, without investigating the subsequent changes in HDL-C level during hospital stay.

Our study has the following advantages over these previous studies: (1) a larger number of hematological indicators included in the multivariate analysis; (2) indicator data not limited to the values obtained at hospital admission and consideration of the effects of subsequent changes in indicator values on the analysis results; and (3) a larger number of patients with severe disease included as subjects.

This study also has some limitations. First, it was a single-center study. Second, the study excluded patients with MAP, which may lead to the inapplicability of the study findings to patients with MAP. Therefore, further prospective studies with a larger sample size are required for validation of our results.

## Conclusions

The lowest HDL-C level may be an independent predictor of progression to SAP in patients with AP admitted to the ICU. The univariate analysis revealed a negative correlation of the lowest HDL-C level with progression to SAP, and multivariate analysis revealed that HDL-C level was an independent protective factor for the aggravation of AP. The lowest HDL-C level may provide clinical guidance for the prediction of SAP onset and development, with an optimum cutoff lowest HDL-C level of 0.545 mmol/L. Considering the lack of research on the relationship between the lowest HDL-C level and SAP, further prospective studies are required for validation of our findings.

## Data availability statement

The raw data supporting the conclusions of this article will be made available by the authors, without undue reservation.

## Ethics statement

The studies involving humans were approved by the Ethics Committee of Shandong Provincial Hospital. The studies were conducted in accordance with the local legislation and institutional requirements. The Ethics Committee waived the requirement of obtaining informed consent because of the retrospective study design.

## Author contributions

QN: Investigation, Methodology, Writing review & editing, Conceptualization. ZY: Writing-original draft, Data curation. PZ: Data curation, Methodology. HJ: Writing review & editing. FL: Investigation, Methodology, Conceptualization. HC: Investigation, Conceptualization, Methodology, Supervision. All authors had full access to all the data in the study and had final responsibility for the decision to submit for publication. All authors contributed to the article and approved the submitted version.
